# Organizational Spirituality: Refining Its Measurement and Exploring Associations with Individual Spirituality at Work and Job Satisfaction in Poland

**DOI:** 10.1007/s10943-025-02431-2

**Published:** 2025-09-07

**Authors:** Marcin Wnuk, Edyta Charzyńska

**Affiliations:** 1 Department of Work and Organizational Psychology, Faculty of Psychology and Cognitive Sciences, Adam Mickiewicz University in Poznań, Szamarzewskiego 89/AB, 60-568 Poznań, Poland; 2 Institute of Psychology, Institute of Pedagogy, Faculty of Social Sciences, University of Silesia in Katowice, Grażyńskiego 53, 40-126 Katowice, Poland

**Keywords:** Organizational spirituality, Workplace spirituality, Religiosity, Validity, Individual spirituality, Job satisfaction

## Abstract

Despite the burgeoning interest in workplace spirituality, there remains considerable room for improvement in its conceptualization and measurement. The purpose of this study was to provide a psychometrically sound and easily implementable measure of organizational spirituality. Additionally, the study aimed to differentiate organizational spirituality from individual spirituality at work by examining its unique contributions to job satisfaction. The study was conducted online with 884 employees in Poland (52.1% women). The Organizational Spiritual Values Scale (OSVS), the “spiritual connection at work” subscale from the Spirit at Work Scale, and a single-item measure of job satisfaction were used. Based on the confirmatory factor analysis, the unidimensional OSVS was shortened from 20 to 10 items to optimize its psychometric properties. The shortened instrument (OSVS-S) demonstrated scalar measurement invariance across gender, age, employees’ religiosity levels, job positions, employment sectors, and organization sizes. Higher levels of organizational spirituality were noted among managers (versus nonmanagers), more religious (versus less religious) employees, and employees working in smaller (versus larger) organizations. Moreover, organizational spirituality predicted job satisfaction even after controlling for individual spirituality at work and sociodemographic variables. This study contributes to the literature by providing a robust measure of organizational spirituality and emphasizes  the need to incorporate workplace spirituality at various levels in future research.

## Introduction

Workplace spirituality is a relatively new area of inquiry that has gained increasing recognition over the past two decades (Barik & Nayak, 2024; Lahmar et al., 2024), though research is still largely concentrated in the United States and other Western countries (Dik et al., 2024; Hassan et al., 2021). Factors driving the growing interest in workplace spirituality include changes in the nature of work, such as the need to cope with heavy demands and high job insecurity, an overall increase in interest in spirituality and spiritual paths, and accumulating evidence of the beneficial role of workplace spirituality in employees’ well-being, performance, and attitudes toward their organizations (for a review of outcomes, see Dik et al., 2024 and Syahir et al., 2025). Additionally, managers and executives have begun to recognize that employees are motivated not only by financial incentives but also by a desire to find meaning in their work, as well as to maintain mental health and well-being (Honiball et al., 2014). This realization has led many leaders to strive to create work environments that support employees’ spiritual growth, harmony, and balance (Kinjerski & Skrypnek, 2004).

The current study aims to improve the available assessment of workplace spirituality at the organizational level. Moreover, it examines the associations between organizational spirituality and job satisfaction while controlling for the effects of individual spirituality at work and sociodemographic variables. The introduction begins with the definition of spirituality applied in this study, followed by a description of workplace spirituality, including its levels, the mechanisms involved in its relationships with work-related outcomes, and the limitations of previous assessments of organizational spirituality.

### Defining Spirituality

A wide variety of definitions and perspectives on spirituality have appeared in the literature (for a systematic review, see De Brito Sena et al., 2021). In the current study, spirituality is treated as the human capacity to experience transcendence (Heszen-Niejodek & Gruszczyńska, 2004; Miller & Thoresen, 2003), understood as going above and beyond the self to adopt a broader perspective, which includes concerns for others and the world, and to experience the all-embracing feeling of being part of a greater whole. Spirituality is also expressed in the innate human drive to search for a sense of purpose and fulfillment in life (Puchalski et al., 2014).

The beneficial role of spirituality for health and well-being is strongly supported by empirical data (for an overview, see Oman & Syme, 2018; Yaden et al., 2022). Spirituality provides individuals with coping strategies, helps shape adaptive cognitive schemas such as a sense of meaning in life, increases social support, enhances self-esteem and self-efficacy, and promotes healthy behaviors and positive emotions such as hope and optimism (Bożek et al., 2020; Charzyńska, 2015; Peres et al., 2018; Wnuk, 2022a, 2023).

Spirituality is often related to religion since, for many individuals, religion serves as a foundation for spiritual development, providing a theological structure that prescribes religious beliefs, values, and practices (Zinnbauer & Pargament, 2005). However, as a universal human phenomenon (Elkins, 2005; Heszen-Niejodek & Gruszczyńska, 2004), spirituality can exist independently of religion. In this form of spirituality, individuals shape their spiritual experience outside a religious framework, for example, through closeness to nature, contemplation, creating or appreciating art, or feeling connected to a greater community based on shared values and a common purpose (De Brito Sena et al., 2021; Jones et al., 2019; Miller & Thoresen, 2003).

### Micro- and Meso-Level Perspectives on Workplace Spirituality

Spirituality has received noticeable attention from scholars in the context of the workplace (Rocha & Pinheiro, 2021). It is essential to first emphasize that the conceptualization of workplace spirituality in the literature encompasses its different levels (Pfaltzgraff-Carlson, 2020; Prabhu et al., 2016). Most studies explore workplace spirituality from an individual, micro-level perspective, calling it “individual spirituality at work,” “spirit at work,” and “employee spirituality” (Kinjerski & Skrypnek, 2004; Wnuk, 2022b). This level of workplace spirituality reflects the sense of a presence of something greater than oneself during the work process, which is connected to the employee’s desire to engage in meaningful work that serves a greater purpose and to establish deep, value-based relationships with coworkers (Kinjerski, 2013). It refers to the individual, intrinsic, and intimate need of employees to bring their spirituality to work and actively involve it in their tasks and duties, giving them a sense of fulfillment (Charzyńska et al., 2021; Hill et al., 2013; Kinjerski & Skrypnek, 2004; Prabhu et al., 2016). Individual spirituality at work serves as a spiritual resource that promotes one’s own well-being and health and can lead to favorable work-related outcomes by enhancing work engagement and organizational commitment, strengthening workplace friendship, trust, and respect, helping create an ethical climate in an organization, and reducing negative attitudes and behaviors toward the job (for details, see a systematic literature review by Dubey & Bedi, 2024).

The other perspective encompasses workplace spirituality at the meso level, also referred to as “organizational spirituality” (Kolodinsky et al., 2008) and “spiritually based organization” (Vasconcelos, 2023). It denotes the organizational culture within a specific workplace that helps its employees experience transcendence through the work process and fosters a sense of connectedness with coworkers who share a common purpose (Hill et al., 2013; Kolodinsky et al., 2008; Pfaltzgraff-Carlson, 2020; Prabhu et al., 2016). In organizations that appreciate and promote spiritual values (such as benevolence, generativity, honesty, humanism, justice, mutuality, openness, receptivity, respect, self-transcendence, and trust; Giacalone & Jurkiewicz, 2003), employees are encouraged and taught to focus on spiritually directed attitudes in the workplace and to follow the spiritual values, rules, codes of conduct, and standards promoted by the organization (Meyer et al., 2020).

The mechanisms underlying the relationship between organizational spirituality and positive work-related outcomes for both employees and organizations include increased job involvement, work engagement, and job satisfaction (Van der Walt, 2018; Van der Walt & de Klerk, 2014b; Van der Walt & Swanepoel, 2015); Watprasong & Varma, 2022). Additional mediating mechanisms may involve enhanced organizational trust and psychological flourishing (Khari & Sinha, 2018, 2021), support for thriving at work (Van der Walt, 2018), the development and maintenance of positive relationships with coworkers, improved emotional and spiritual competence, and the fostering of a sense of wholeness and harmony (see Honiball et al., 2014). Moreover, as demonstrated in a study by Van der Walt and Steyn (2019) involving project managers, organizational spirituality may also influence ethical behavior by promoting responsibility and fairness (see also Honiball et al., 2014).

It should be emphasized at this point that the terms workplace spirituality at the individual and organizational levels have sometimes been used interchangeably in different contexts, causing confusion and ambiguity (Kolodinsky et al., 2008; Rocha & Pinheiro, 2021). Moreover, some research combines both levels of workplace spirituality by drawing conclusions about organizational spirituality based on aggregated individual-level data, which is often insufficiently justified (Pfaltzgraff-Carlson, 2020).

### Measurement of Organizational Spirituality

In the vast majority of studies, workplace spirituality has been considered from an individual perspective (Dubey & Bedi, 2024; Kinjerski & Skrypnek, 2004; Wnuk, 2024). Research exploring organizational spirituality as a work environment that incentivizes employees’ spiritual development and the expression of spiritual values at work is still relatively rare (Rocha & Pinheiro, 2021; Vasconcelos, 2023), despite its beneficial impacts on work-related outcomes (Kolodinsky et al., 2008; Mhatre & Mehta, 2023; Rocha & Pinheiro, 2021; Van der Walt & de Klerk, 2014b). One of the challenges in studying organizational spirituality is the scarcity of measures developed to assess this construct (Pawar, 2023; Prabhu et al., 2016). The problem was partially addressed by Kolodinsky et al. (2008) when they developed the Organizational Spiritual Values Scale (OSVS), which assesses employees’ perceptions of the spiritual values exhibited by their organization. The OSVS is a distinctly secular measure (free of any religious content), with items referring to a specific organizational culture where employees share common spirituality-based values, are encouraged to seek meaning through work, and nurture meaningful relationships with coworkers.

Using the OSVS, Kolodinsky et al. (2008) found that in samples of full-time workers taking graduate coursework, organizational spirituality was positively related to attitudinal and attachment-related outcomes such as job involvement, work rewards satisfaction, and organizational satisfaction, and was negatively related to organizational frustration. A study by Van der Walt (2018) conducted among employees in South Africa showed that organizational spirituality measured with the OSVS was related to affective-motivational states such as work engagement and thriving at work. In another study using the OSVS (Van der Walt & Klerk, 2014a), among the biographic variables measured (i.e., respondents’ gender, age, education level, ethnicity, strength of religious conviction, and employing organization), differences in levels of organizational spirituality were noted for respondents’ age (with the 30–40 year age group experiencing the highest levels, followed by the 41–62 year age group, and lastly, the 19–29-year age group) and employing organization (with employees in educational organizations reporting higher levels of organizational spirituality than those in healthcare organizations).

Although the OSVS has gained some interest and has been used in several studies, its main drawback lies in the limited research on its psychometric properties. Specifically, although its reliability has been often examined (Dean, 2019; Pawar, 2014), its factorial validity has been scarcely tested. Some studies used principal factor analysis to examine the factorial structure of the OSVS, which supported the unidimensionality of the measure after removing the sole negatively worded item (i.e., Item 13) (Van der Walt & de Klerk, 2014b; Van der Walt & Steyn, 2019). Moreover, when confirmatory factor analysis (CFA) was used, the model fit for the OSVS was found to be suboptimal (Van der Walt & de Klerk, 2014a, 2015). Additionally, although the OSVS is a unidimensional measure, it consists of as many as 20 items, raising concerns about the potential redundancy of some questions.

### Current Study

Given the growing interest in organizational spirituality and the need for a valid and reliable measure, this study aims to test the psychometric properties of the OSVS and refine the scale if necessary. Additionally, to ensure that organizational spirituality is understood consistently across diverse employee groups, we will examine the measurement invariance of the OSVS across gender, age, religiosity level, job position, employment sector, and organization size. Regarding group differences in latent means, we hypothesize that employees with higher levels of religiosity and those in managerial positions will perceive higher levels of organizational spirituality compared to employees with lower levels of religiosity or those in nonmanagerial roles (Hypothesis 1 [H1]). Religious employees may be more inclined to seek spiritual values within their organizations (particularly in highly religious countries such as Poland), which can influence their perceptions of organizational spirituality (Mazur, 2021). Managers may report higher levels of organizational spirituality than nonmanagers, as they often play a key role in shaping the spiritual climate of the organization by setting standards, modeling values, and establishing practices that influence the broader organizational culture (Fry et al., 2017).

The final aim of the current study is to explore the associations between organizational spirituality, workplace spirituality at the individual level, and job satisfaction. Spiritual employees may actively seek workplaces that align with and support their spiritual values (Kristof, 1996; Kristof-Brown et al., 2005). Moreover, organizations that promote spiritual values may create a climate that enhances workplace spirituality at the individual level (Meyer et al., 2020). Based on these premises, we expect that individual spirituality at work and organizational spirituality are positively related (H2). Furthermore, we hypothesize that organizational spirituality predicts unique variance in job satisfaction above and beyond workplace spirituality at the individual level and sociodemographic variables, thereby supporting the incremental validity of the measure (H3).

## Methods

### Sample

The sample consisted of 884 Polish employees (52.1% women). The sociodemographic and work-related characteristics of the participants are presented in Table 1. The mean age of the participants was 42.3 years (*SD* = 12.0). Most participants were married (55.3%), held a higher education (56.7%), and identified as Catholic (57.1%). On average, the participants had one child (*M* = 1.15, *SD* = 1.1). Most participants were employed full-time (84.1%), worked in the private sector (64.5%), and worked in organizations with at least 50 employees (60.3%). The average work experience was 18.0 years (*SD* = 11.7), and the average tenure with the current employer was almost nine years (*M* = 8.9, *SD* = 9.0). On average, participants declared working 39.2 h per week (*SD* = 10.1). Approximately one quarter (23.9%) of the participants held managerial positions.

**Table 1:** Participant demographic characteristics (N = 884)

Variable	*n* (%)
*Gender*	
Men	422 (47.7)
Women	461 (52.1)
Other	1 (0.1)
*Age (in years)*	42.31 (11.99)^a^
*Marital status*	
Single	135 (15.3)
Married	489 (55.3)
Informal relationship	197 (22.2)
Widower	13 (1.5)
Divorced	42 (4.8)
Separated	8 (0.9)
*Number of children*	1.15 (1.07)^a^
*Education level*	
Primary	2 (0.2)
Lower secondary	2 (0.2)
Vocational	54 (6.1)
High school	325 (36.8)
Higher	478 (54.1)
Ph.D	23 (2.6)
*Religion*	
Catholicism	505 (57.1)
Jehovah's Witnesses	6 (0.7)
Greek	5 (0.6)
Protestantism	3 (0.3)
Other denominations	8 (0.9)
Without a particular religious denomination	26 (2.9)
Agnosticism	36 (4.1)
Atheism	74 (8.4)
I prefer not to respond to this question	221 (25.0)
*Work experience (in years)*	18.03 (11.74)^a^
*Monthly income before taxes*	
Less than 2,500 zl	42 (4.7)
2,500–3,999 zl	181 (20.5)
4,000–5,499 zl	205 (23.2)
5,500–6,999 zl	154 (17.4)
7,000–8,499 zl	77 (8.7)
8,500–9,999 zl	51 (5.8)
10,000 zl and more	59 (6.7)
I prefer not to respond to this question	115 (13.0)
*Working hours per week*	39.24 (10.13)^a^
*Form of employment*	
Full-time contract	744 (84.1)
Part-time contract	37 (4.2)
Civil law contract	97 (11.0)
Other	6 (0.7)
*Work duration for the current employer (in years)*	8.87 (8.97)^a^
*Employment sector*	
Public	296 (33.5)
Private	570 (64.5)
Non-governmental organization	18 (2.0)
*Organization size*	
1–9 employees	117 (13.2)
10–49 employees	234 (26.5)
50–249 employees	219 (24.8)
250 employees and more	314 (35.5)
*Managerial position*	
No	673 (76.1)
Yes, a lower-level manager	100 (11.3)
Yes, a middle-level manager	72 (8.2)
Yes, a higher-level manager	39 (4.4)

^a^For continuous variables, means and standard deviations (in parentheses) are presented

### Procedure

The study was conducted using the Ariadna research panel, which operates in Poland. The following inclusion criteria were used: (1) being an adult, (2) living and working in Poland, and (3) having at least three years of professional experience. The exclusion criteria included being self-employed. Participants were offered points for participation, which could be exchanged for prizes after collecting a given number of points.

The study was conducted in accordance with the Declaration of Helsinki. The project received ethical approval from the Ethics Committee at the University of Silesia in Katowice, Poland (KEUS417/10.2023). The participants were informed that responses to the survey were anonymous, and that they could withdraw from the study at any point, without giving any reasons and without any consequences. Each participant provided online informed consent before starting the survey. No personal data were collected.

### Measures

#### Organizational Spirituality

The original 20-item OSVS scale (Kolodinsky et al., 2008) is based on the Human Spirituality Scale (HSS; Wheat, 1991). To create the OSVS, the items from the HSS were rephrased into statements designed to assess individuals’ perceptions of spiritual values manifested by their organization. The measure is unidimensional, representing a single factor labeled “organizational spirituality.” The items (of which 19 are positively worded and one is negatively worded) are scored on a five-point Likert scale (1 = “completely false,” 5 = “completely true”).

The translation and validation of the OSVS followed the standard procedure described by Koenig and Al Zaben (2021) to ensure conceptual equivalence to the original scale and cultural relevance for the target population. The preparation of the Polish version of the OSVS included obtaining the author’s permission and translating the original English version following the best practices outlined by the Professional Society for Health Economics and Outcomes Research (Wild et al., 2005). A step-by-step description of the translation process applied in this study is provided in Appendix 1. The validation of the translated scale, which is described below, included confirmatory factor analysis (CFA) to assess factor validity, item reduction based on the CFA results, reexamination of the revised model, and evaluation of the scale’s reliability, construct validity, and incremental validity (Koenig & Al Zaben, 2021).

#### Workplace Spirituality at the Individual Level

The subscale “spiritual connection at work” from the Polish version (Charzyńska et al., 2025) of the Spirit at Work Scale (SAWS; Kinjerski, 2013) was used to assess the participants’ workplace spirituality at the individual level. This subscale is one of the three within the Polish version of the SAWS that specifically assesses pure individual spirituality at work, distinguishing it from the two other subscales, i.e., meaningful work and the sense of community at work, which reflect positive psychological characteristics such as purpose in life, meaning, and connection. These latter aspects can lead to distorted (usually inflated) relationships between spirituality and outcomes (Charzyńska et al., 2025; see also Koenig & Carey, 2024, 2025); therefore, only the “spiritual connection at work” subscale (e.g., “I receive inspiration or guidance from a Higher Power about my work”) was analyzed in the current study. The three items of this scale are rated on a six-point Likert scale (1 = “completely untrue” to 6 = “completely true”). The reliability of the “spiritual connection at work” subscale in the current study, as measured by Cronbach’s alpha, was 0.94.

#### Job Satisfaction

Job satisfaction was measured with a single item developed by Dolbier et al. (2005): “Taking everything into consideration, how do you feel about your job as a whole?” The item is rated using a seven-item Likert scale (1 = “not at all satisfied” to 7 = “very satisfied”). A single item highly correlated with the 15-item measure of job satisfaction (Dolbier et al., 2005). Moreover, research has demonstrated that this short measure represents a reliable and valid operationalization of job satisfaction (Weiss et al., 2022).

#### Religiosity

Religiosity was measured using a single question: “To what extent do you consider yourself to be a religious person?” The participants responded to this question using a seven-point Likert scale (1 = “not at all religious” to 7 = “very religious”). A single-item measure of religiosity has been used in many studies, which supported its reliability and validity (Abdel-Khalek, 2007).

### Statistical Analyses

We initially calculated the descriptive statistics for all OSVS items. Next, a CFA with a maximum likelihood with robust standard errors (MLR) estimator was performed to examine the factorial validity of the original version of the OSVS. In line with theoretical premises and previous studies, the OSVS items were expected to load onto a single latent construct labeled “organizational spirituality.” The following criteria were used to assess model fit: relative χ^2^ (χ^2^/df), comparative fit index (CFI), Tucker-Lewis Index (TLI), root mean square error of approximation (RMSEA), and standardized root mean square residual (SRMR). The values of the relative χ^2^ ≤ 3 (or ≤ 5), CFI and TLI ≥ 0.95 (or ≥ 0.90), RMSEA ≤ 0.06 (or ≤ 0.10), and SRMR ≤ 0.08 (or ≤ 0.10) suggest good (or acceptable) model fit, respectively (Kline, 2016; Wang & Wang, 2012).

After examining the model fit for the original OSVS, the item-reduction procedure was initiated. When removing items, the values of the modification indices (MIs) were considered first. Specifically, the largest MI value for the pair of items was detected, and the wording of those items was compared to check if their meaning was similar (Bandalos, 2021). Moreover, their standardized factor loadings were compared to select the better item. This procedure was repeated until only the best-fitting items remained, ensuring both the content validity of the measure and its good psychometric properties. The model fit of the short version of the OSVS (called OSVS-S) was assessed using the same criteria applied to the original OSVS.

Once the structure of the OSVS-S was established, the measurement invariance across gender, age, employees’ religiosity levels, job positions, employment sectors, and organization sizes was tested using multiple-group CFA (MGCFA). This was done to ensure that OSVS-S scores could be meaningfully compared between groups. For each variable, three models were tested: (1) a configural model, in which the same factor structure (i.e., the number of factors and corresponding items) applies to all groups, but model parameters (loadings and intercepts) are freely estimated; (2) a metric model, in which the additional constraints are imposed to ensure equality of factor loadings across groups; and (3) a scalar model, in which additional constraints are imposed to ensure equality of intercepts across groups (Chen, 2007).

To evaluate the measurement invariance of the OSVS-S, changes (Δ) in CFI, TLI, RMSEA, and SRMR between the subsequent (i.e., more constrained) models were compared. The measurement invariance of a more constrained model was considered established when no significant deterioration in model fit was detected across the subsequent models. Specifically, the changes in the alternative fit indices were deemed negligible if they met the following criteria: ΔCFI and ΔTLI ≤ –0.010, ΔRMSEA < 0.015, ΔSRMR < 0.030 (for loading invariance), and ΔSRMR < 0.010 (for intercept invariance) (Chen, 2007; Cheung & Rensvold, 2002).

After testing the measurement invariance, latent mean comparisons were conducted across gender, age, employees’ religiosity levels, job positions, employment sectors, and organization sizes. The critical ratio value, calculated by dividing the parameter estimate by the standard error, was used to assess the latent mean differences. When the absolute value of the critical ratio exceeds 1.96, the difference in latent means between the comparison group and the reference group is statistically significant at *p* < 0.05.

In the next step of the analysis, we calculated the reliability of the OSVS-S using Cronbach’s alpha coefficient. We also examined the convergent validity of the measure by calculating its average variance extracted (AVE) and composite reliability (CR). The AVE ≥ 0.5 and CR ≥ 0.7 values indicate good convergent validity of the measure (Fornell & Larcker, 1981).

Next, we examined the associations between organizational spirituality and various sociodemographic and work-related variables, including gender, age, religiosity, job position, employment sector, organization size, individual spirituality at work, and job satisfaction. To do so, we used Pearson correlations for continuous variables, point-biserial correlations for continuous and binary variables, and Spearman correlations for continuous and ordinal variables.

In the last step of the analysis, after checking the relevant assumptions (i.e., linearity, normality, independence of errors, and independence of predictors), a hierarchical multiple regression analysis was performed to assess whether organizational spirituality contributes to job satisfaction above and beyond the contribution of individual spirituality at work and sociodemographic and work-related variables. When selecting control variables, the findings of previous studies on job satisfaction were considered (see Andrade et al., 2019; Colin-Chevalier et al., 2024). As a result, the following variables were entered into each step of the hierarchical multiple regression analysis: (1) gender and age, (2) job position, (3) individual spirituality at work, and (4) organizational spirituality. All calculations were conducted using IBM SPSS (IBM Corp. Released 2021) and MPlus, version 8.0 (Muthén & Muthén, 2017).

## Results

### Factorial Structure of the OSVS

There were no missing values for the variables used in the study. Descriptive statistics for the OSVS are presented in Appendix 2. The means ranged from 2.28 (Item 2) to 3.28 (Items 13 and 20). The results of the CFA for the 20-item OSVS showed that the model fit was unacceptable, as indicated by the values of the model fit criteria (χ^2^(170) = 1,178.86, χ^2^/df = 6.93, CFI = 0.878, TLI = 0.864, RMSEA = 0.082 (90% CI 0.078, 0.086), SRMR = 0.051). For the negatively worded Item 13, the standardized loading was –.11. For the rest of the items, the standardized loadings ranged from 0.57 (Item 1) to 0.87 (Item 9; see Appendix 2).

To improve the psychometric properties of the measure, Item 13 was first removed from the analysis. Next, the CFA was rerun, and the modification indices (MIs) were examined. The content of the items was then compared to identify potentially redundant items, and their standardized factor loadings were also reviewed; this process was repeated as necessary. A detailed description of the item comparison and removal process is provided in Appendix 3. Ultimately, the 10-item version of the OSVS (named the OSVS-S) was established and subjected to further analyses.

### Final Version of the OSVS-S

**Fig. 1:**
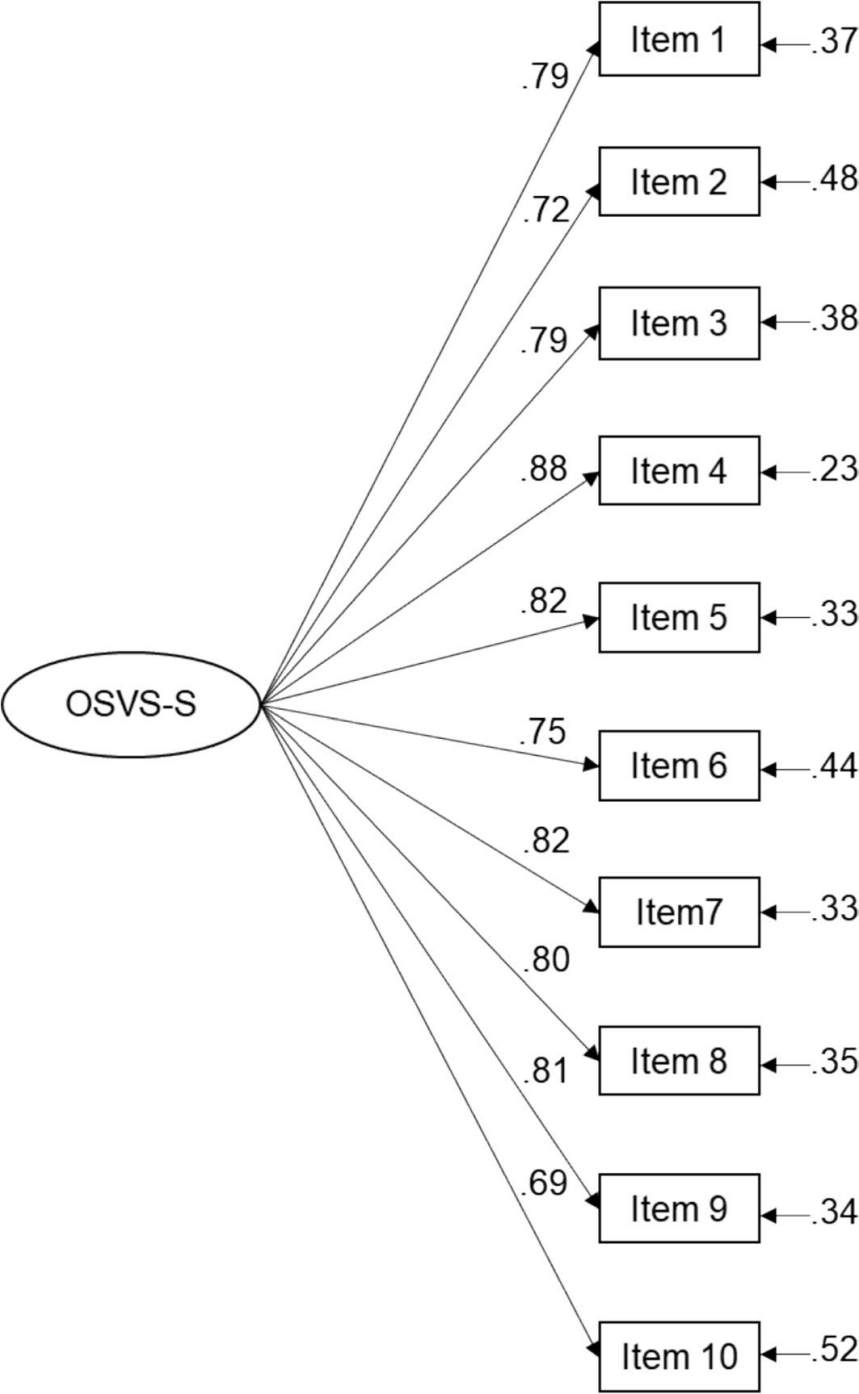
Confirmatory factor analysis of the short version of the Organizational Spiritual Values Scale (OSVS-S) with standardized loadings

The OSVS-S demonstrated an excellent model fit (χ^2^(35) = 87.66, χ^2^/df = 2.50, CFI = 0.984, TLI = 0.980, RMSEA = 0.041 (90% CI 0.031, 0.052, SRMR = 0.021). The items and response categories for the English and Polish versions of the OSVS-S are presented in Table 2. The full versions of the OSVS-S are included in Appendix 4. Figure 1 and Appendix 5 present the standardized loadings and descriptive statistics for the OSVS-S. The factor loadings were high, ranging from 0.69 to 0.88.

**Table 2:** Items and response options for the short version of the Organizational Spiritual Values Scale (OSVS-S)

Items of the short version of the Organizational Spiritual Values Scale (OSVS-S)	
English	Polish (Polski)
1. The organization values the relationship among everyone who works here	1. Ta organizacja/firma docenia relacje, jakie się tworzą między pracownikami
2. Being truthful is important to a successful life in this organization	2. Aby odnieść sukces w tej organizacji/firmie, ważna jest prawdomówność
3. This organization is sensitive to the pain and suffering of others	3. W tej organizacji/firmie widać wrażliwość na ból i cierpienie innych
4. It is important to this organization that employees are whole and complete people	4. Ta organizacja/firma dba o to, by pracownicy byli ludźmi spełnionymi i mieli poczucie kompletności
5. In this organization, all forms of life are valuable	5. W tej organizacji/firmie ceni się wszystkie formy życia
6. There is an overall sense of sadness when someone in this organization is in pain	6. Jeżeli ktoś w tej organizacji/firmie cierpi, to panuje ogólne poczucie smutku
7. This organization promotes health and inner peace	7. Ta organizacja/firma promuje zdrowie i spokój wewnętrzny
8. The organization encourages us to put the interests of others before our own when making a decision	8. Ta organizacja/firma zachęca nas do tego, by przy podejmowaniu decyzji dbać przede wszystkim o dobro innych
9. In this organization we are encouraged to actively seek a sense of purpose in our lives	9. W tej organizacji/firmie jesteśmy zachęcani, by aktywnie poszukiwać celu swojego życia
10. We are encouraged to mentor and help new people entering the organization	10. Jesteśmy zachęcani, by wspierać nowe osoby dołączające do organizacji/firmy i im pomagać
Response options: 1 = Mostly false, 2 = Somewhat false, 3 = Somewhat true, 4 = Mostly true, 5 = Completely true	Response options (opcje odpowiedzi): 1 = W ogóle nieprawdziwe, 2 = Raczej nieprawdziwe, 3 = Ani prawdziwe, ani nieprawdziwe, 4 = Raczej prawdziwe, 5 = Zdecydowanie prawdziwe

The OSVS-S items were derived from the 20-item Organizational Spiritual Values Scale (Kolodinsky et al., 2008)

### Measurement Invariance and Latent Mean Differences

The results of the measurement invariance testing are presented in Table 3. The changes in the model fit indices for the more constrained models supported the scalar measurement invariance for all the variables analyzed. This result indicates that the latent mean values obtained for the analyzed subgroups can be compared meaningfully.

**Table 3:** Goodness of fit indices and model comparisons for measurement invariance models

Model		χ^2^	*df*	CFI	TLI	RMSEA (90% CI)	SRMR	Model comparison	ΔCFI	ΔTLI	ΔRMSEA	ΔSRMR
*Gender*												
Men (*n* = 422)		89.60	35	0.967	0.957	0.061 (0.045, 0.077)	0.030					
Women (*n* = 461)		65.71	35	0.984	0.979	0.044 (0.027, 0.060)	0.022					
	(A) Configural	158.20	70	0.975	0.967	0.053 (0.042, 0.065)	0.026					
	(B) Metric	179.27	79	0.971	0.967	0.054 (0.043, 0.064)	0.041	B vs. A	−0.004	0.000	0.001	0.015
	(C) Scalar	191.77	88	0.970	0.969	0.052 (0.042, 0.062)	0.043	C vs. B	−0.001	0.002	−0.002	0.002
*Age (in years)*												
18–34 (*n* = 276)		47.79	35	0.987	0.983	0.036 (0.000, 0.060)	0.030					
35–44 (*n* = 253)		47.81	35	0.986	0.982	0.038 (0.000, 0.063)	0.026					
45–54 (*n* = 204)		78.81	35	0.955	0.943	0.078 (0.055, 0.101)	0.033					
55 and more (*n* = 151)		63.92	35	0.963	0.953	0.074 (0.044, .102)	0.033					
	(A) Configural	237.23	140	0.974	0.966	0.056 (0.044, 0.068)	0.030					
	(B) Metric	271.40	167	0.972	0.969	0.053 (0.041, 0.064)	0.044	B vs. A	−0.002	0.003	−0.003	0.014
	(C) Scalar	3230.75	194	0.965	0.967	0.055 (0.044, .065)	0.045	C vs. B	−0.007	−0.002	0.002	0.001
*Religiosity level*												
Less religious (*n* = 408)		133.79	35	0.938	0.920	0.083 (0.069, 0.098)	0.041					
More religious (*n* = 316)		84.03	35	0.953	0.939	0.067 (0.048, 0.085)	0.039					
	(A) Configural	214.25	70	0.944	0.928	0.075 (0.064, 0.087)	0.040					
	(B) Metric	234.21	79	0.940	0.932	0.074 (0.063, 0.085)	0.047	B vs. A	−0.004	0.004	−0.001	0.007
	(C) Scalar	263.83	88	0.932	0.930	0.074 (0.064, 0.085)	0.049	C vs. B	−0.008	−0.002	0.000	0.002
*Job position*												
Nonmanagers (*n* = 673)		218.64	35	0.927	0.906	0.088 (0.077, 0.100)	0.042					
Managers (*n* = 211)		27.05	35	1.000	1.000	0.000 (0.000, 0.030)	0.025					
	(A) Configural	245.78	70	0.946	0.930	0.075 (0.065, 0.086)	0.039					
	(B) Metric	264.89	79	0.942	0.934	0.073 (0.063, 0.083)	0.045	B vs. A	−0.004	0.004	−0.002	0.006
	(C) Scalar	282.54	88	0.940	0.938	0.071 (0.062, 0.080)	0.046	C vs. B	−0.002	0.004	−0.002	0.001
*Employment sector*												
Public (*n* = 296)		77.27	35	0.967	0.957	0.064 (0.045, 0.083)	0.030					
Private (*n* = 570)		88.19	35	0.976	0.969	0.052 (0.038, 0.065)	0.026					
	(A) Configural	165.18	70	0.973	0.965	0.056 (0.045, 0.067)	0.027					
	(B) Metric	179.43	79	0.971	0.967	0.054 (0.044, 0.065)	0.033	B vs. A	−0.002	0.002	−0.002	0.006
	(C) Scalar	202.59	88	0.967	0.966	0.055 (0.045, 0.065)	0.035	C vs. B	−0.004	−0.001	0.001	0.002
*Organization size*												
1–9 (*n* = 117)		56.71	35	0.968	0.959	0.073 (0.035, 0.106)	0.034					
10–49 (*n* = 234)		52.61	35	0.983	0.979	0.046 (0.016, 0.071)	0.028					
50–249 (*n* = 219)		56.53	35	0.973	0.965	0.053 (0.025, 0.078)	0.033					
250 and more (*n* = 314)		60.91	35	0.979	0.972	0.049 (0.027, 0.069)	0.029					
	(A) Configural	226.84	140	0.977	0.970	0.053 (0.040, 0.065)	0.030					
	(B) Metric	256.27	167	0.976	0.974	0.049 (0.037, 0.061)	0.042	B vs. A	−0.001	0.004	−0.004	0.012
	(C) Scalar	319.12	194	0.967	0.969	0.054 (0.043, 0.064)	0.048	C vs. B	−0.009	−0.005	0.005	0.006

*df* = degrees of freedom, CFI = comparative fit index, TLI = Tucker-Lewis index, RMSEA = root mean square error of approximation, CI = confidence interval, SRMR = standardized root mean residual, Δ = change between a less restricted and a more restricted model. Data from a participant who chose the response “other” on the item concerning gender was not included in this analysis. Participants who responded from “1” to “3” to the religiosity item were classified as “less religious,” whereas those participants who responded from “5” to “7” to this item were classified as “more religious.” For the employment sector, due to the low number of non-governmental organization employees (*n* = 18), measurement invariance testing included only the public and private sectors

Table 4 presents the results of the latent mean comparison for all variables included in the analysis. More religious employees, managers, and those working in micro organizations reported higher levels of organizational spirituality compared to less religious employees, nonmanagers, and employees working in organizations with 50–249 employees or those in organizations with 250 or more employees. No significant differences in levels of organizational spirituality were observed between genders or employment sectors, or across age groups.

**Table 4:** Latent mean differences

Group	Mean estimate	Standard error	Critical ratio	*p*
*Gender*				
Women (vs. men)	−0.01	0.07	−0.12	0.908
*Age (in years)*				
35–44 (vs. 18–34) 45–54 (vs. 18–34) 55 and more (vs. 18–34)	0.07 0.09 0.16	0.09 0.09 0.11	0.75 0.96 1.47	0.452 0.337 0.142
*Religiosity level*				
More religious (vs. less religious)	0.49	0.08	6.14	< 0.001
*Job position*				
Managers (vs. nonmanagers)	0.33	0.08	4.09	< 0.001
*Employment sector*				
Private (vs. public) sector	−0.01	0.08	−0.10	0.922
*Organization size*				
10–49 (vs. 1–9) employees	−0.20	0.12	−1.63	−0.104
50–249 (vs. 1–9) employees	−0.31	0.12	−2.61	0.009
250 and more (vs. 1–9) employees	−0.31	0.11	−2.71	0.007

Dummy coding was used for gender (0 = men, 1 = women), religiosity level (0 = less religious, 1 = more religious), job position (0 = nonmanagers, 1 = managers), and employment sector (0 = public, 1 = private). Participants who responded from “1” to “3” to the religiosity item were classified as “less religious,” whereas those who responded from “5” to “7” were classified as “more religious.” Men, employees aged 18–34, less religious employees, nonmanagers, public sector employees, and employees working in micro organizations (1–9 employees) were treated as reference groups

### Reliability and Convergent Validity

The excellent internal consistency of the OSVS-S was indicated by Cronbach’s α value of 0.94. Moreover, the AVE (.62) and CR (.94) values indicated good convergent validity of the measure.

### Correlations with Sociodemographics and Work-Related Variables

Being older (*r* = 0.07, *p* = 0.041), more religious (*r* = 0.24, *p* < 0.001), and holding a managerial position (*r* = 0.14, *p* < 0.001) were positively related to higher perceived levels of organizational spirituality (see Appendix 6). Additionally, working at smaller organizations was associated with higher perceived organizational spirituality, as indicated by a negative correlation with organization size (rho = –0.10, *p* = 0.007). Moreover, organizational spirituality was positively related to individual spirituality at work (*r* = 0.42, *p* < 0.001) and job satisfaction (*r* = 0.53, *p* < 0.001). Notably, the relationship between individual spirituality at work and religiosity (*r* = 0.68, *p* < 0.001) was much stronger than that between organizational spirituality and religiosity (*r* = 0.24, *p* < 0.001; *z* = −8.39, *p* < 0.001). By contrast, the relationship between organizational spirituality and job satisfaction (*r* = 0.53,* p* < 0.001) was twice as strong as that between individual spirituality at work and job satisfaction (*r* = 0.25, *p* < 0.001; *z* = −3.22, *p* < 0.001).

### Predictive Role of Organizational Spirituality in Job Satisfaction

To ensure that no multicollinearity problem exists in our data, we used the variance inflation factor (VIF) and tolerance value. There were no predictors with values of VIF exceeding 10 and with values of tolerance lower than 0.1 (maximum VIF of 1.23 and minimum tolerance of 0.81 were noted for indivdiual spirituality at work). These results suggest that the multicollinearity issue was not likely to occur in our data (Marcoulides & Raykov, 2019).

Table 5 presents the results of the hierarchical multiple regression analysis examining the predictive role of organizational spirituality on job satisfaction. After controlling for other variables, organizational spirituality emerged as a significant predictor of job satisfaction (β = 0.51; *p* < 0.001). The previously significant relationship between individual spirituality at work and job satisfaction became non-significant once organizational spirituality was introduced into the regression model.

**Table 5:** Results of hierarchical multiple regression analysis

Predictor	Job satisfaction			
	β	*p*	∆*R*^**2**^	*p*
Step 1			0.016	< 0.001
Gender	−0.02	0.586		
Age	**0.12**	** < 0.001**		
Step 2			0.012	0.001
Gender	0.00	0.981		
Age	**0.11**	**0.001**		
Job position	**0.11**	**0.001**		
Step 3			0.048	< 0.001
Gender	−0.01	0.851		
Age	**0.09**	**0.007**		
Job position	**0.08**	**0.012**		
Individual spirituality at work	**0.22**	** < 0.001**		
Step 4			0.210	< 0.001
Gender	−0.02	0.500		
Age	**0.08**	**0.007**		
Job position	0.04	0.181		
Individual spirituality at work	0.02	0.606		
Organizational spirituality	**0.51**	** < 0.001**		

β = standardized regression coefficient; ∆*R*^2^ = change in *R*^2^ value between the steps. Significant standardized regression coefficients and their corresponding *p*-values were bolded. Dummy coding was used for gender (0 = men, 1 = women) and job position (0 = nonmanagers, 1 = managers). Data from a participant who chose the response “other” on the item concerning gender was not included in this analysis. *N* = 883

## Discussion

Although workplace spirituality plays a significant role in employees’ well-being and performance, the topic of organizational spirituality is still underexplored (Mhatre & Mehta, 2023; Rocha & Pinheiro, 2021). To some extent, this is caused by ambiguity in conceptualizing workplace spirituality, which manifests in confusing its different levels (Kolodinsky et al., 2008; Pfaltzgraff-Carlson, 2020; Rocha & Pinheiro, 2021). Moreover, providing researchers with a psychometrically sound and short measure of organizational spirituality is crucial to advancing research on spiritual culture at work (Dik et al., 2024).

In relation to the above, the main purpose of this study was to prepare a sound measure of workplace spirituality by testing the psychometric properties of the OSVS and refining the scale if necessary. Quite surprisingly, most previous studies have lacked a thorough examination of OSVS’s properties, leaving the scale’s factorial validity insufficiently explored. Moreover, since the original 20-item version of the OSVS is considered unidimensional, we expected some items to be redundant, and applying Occam’s razor would be advisable. Therefore, in the current study, the measure was shortened by comparing items with similar meanings as indicated by the values of MIs; moreover, factor loadings were taken into account. Shortening the scale resulted in a one-factor solution consisting of 10 items with an excellent fit to the data. The high internal consistency of the OSVS-S was supported by the value of Cronbach’s α, which was 94. The AVE and CR values further indicated high convergent validity of the OSVS-S.

The scalar measurement invariance of the OSVS-S was supported for gender, age, employees’ religiosity levels, job positions, employment sectors, and organization sizes. To the authors’ knowledge, no previous studies have examined whether the items of the OSVS are equivalent across various subgroups, and thus whether their means can be meaningfully compared.

The latent mean comparison showed that the level of organizational spirituality was higher among more religious employees (versus less religious) and managers (versus nonmanagers) (H1 supported). Although neither workplace spirituality at the individual level nor at the organizational level should be equated with religion (Van der Walt & de Klerk, 2014a), the results of the current study suggest that more religious employees may be more inclined to perceive organizational climate as more spiritual. Importantly, the relationship between individual spirituality at work and religiosity was considerably stronger than that between organizational spirituality and religiosity, indicating that personal spiritual orientation is more closely tied to religious beliefs than are perceptions of the organizational culture.

Given that religion enhances social ties and helps build and maintain relationships (Lim & Putnam, 2010; Nezlek, 2021), religious employees may support one another within the organization and foster a shared purpose based on common religious values and beliefs, which can influence their perception of organizational spirituality. This effect may be more pronounced or specific to highly religious countries such as Poland, where religiosity is often regarded as a primary path to nurturing personal spirituality (Mazur, 2021; Wnuk, 2018). This area merits further investigation in future research.

A higher level of organizational spirituality observed among managers compared to nonmanagers can be understood from the perspective of their role as representatives of the organization, who are responsible for implementing and promoting spiritual values and therefore may identify more strongly with these values than other employees (Levinson, 1965; Reave, 2005). It is also possible that managers overestimate the actual level of organizational spirituality in their workplace because they are actively involved in cultivating spiritual values among staff. As a result, they might inaccurately perceive the extent to which other employees share the standards and practices promoted by management, potentially engaging in wishful thinking.

Though this was not hypothesized, the perceived level of organizational spirituality was higher in employees working in small organizations compared to those in middle or large organizations. This finding suggests that in smaller businesses, spiritual values may be more transparent and easier to implement successfully in the workplace. A flatter organizational structure with less bureaucracy, fewer procedures, and fewer formal instructions can more effectively facilitate the creation of a work environment focused on meaningful work and on supporting employees in their efforts to find purpose in their roles and establish profound relationships with coworkers who share similar values and goals (see Driscoll et al., 2019).

As expected, individual spirituality at work and organizational spirituality were positively related (H2 supported), with a moderate correlation noted (*r* = 0.42; *p* < 0.001). Organizations that cultivate spiritual values and promote spiritual growth may create a climate that enhances individual spirituality at work (Fry et al., 2017**).** It is also plausible that more spiritually oriented individuals are drawn to organizations that align more closely with their personal values and beliefs, seeking environments where their sense of connection to something greater than the self is supported and reinforced **(**Kristof, 1996; Kristof-Brown et al., 2005). Nevertheless, the moderate strength of the correlation between these two variables indicates that, although related, they represent distinct levels of workplace spirituality. As such, they should not be conflated and ought to be included together in models to disentangle their specific impact on work-related outcomes.

This was further supported by the results of hierarchical multiple regression analysis, where organizational spirituality significantly explained job satisfaction after controlling for individual spirituality at work and sociodemographic variables (H3 supported). Notably, when organizational spirituality was added to the regression model in the final step, the previously significant effect of individual spirituality at work disappeared. This finding suggests that the broader organizational environment, aimed at promoting spiritual values, may play a more decisive role in shaping how satisfied employees feel than their personal spiritual experiences at work.

This result underscores the importance of fostering a spiritually supportive organizational culture, and not only focusing on individual spirituality. Moreover, the results of the hierarchical multiple regression analysis highlight the practical relevance of the OSVS-S and suggest that interventions at the organizational level could lead to broader and more sustained improvements in employee outcomes than those focused solely on individual spirituality at work.

Lastly, when discussing the new or validated scale in the field of the psychology of spirituality and religion, the issue of the risk of contaminated scales should be raised. In recent years, Bambling (2024) and Koenig and Carey (2024, 2025) have made significant contributions to the psychology of religion and spirituality literature by highlighting the issue of contaminated scales, where items intended to assess spirituality also include mental and social health indicators. This contamination may result in distorted estimates of the relationship between spirituality and its potential outcomes.

However, this concern does not apply to the OSVS-S, which assesses a meso-level factor capturing a workplace culture that values spiritual principles, promotes spiritually driven behavior, and upholds specific spiritual values, rules, and standards of conduct. Notably, such an organizational culture may enhance employee well-being. Yet, this is not always the case. According to person–organization fit theory (Kristof, 1996; Kristof-Brown et al., 2005), individuals with certain characteristics (e.g., high levels of Dark Triad traits; Jonason & Webster, 2010) may experience a value-based misfit when working in spiritually oriented organizations that emphasize interconnectedness with something greater than the self, communal values, compassion, and a sense of shared purpose. This misalignment may result in negative outcomes, such as reduced job satisfaction or increased psychological strain. These reflections emphasize the importance of investigating organizational spirituality as a meso-level construct that potentially moderates the relationship between individual characteristics and work-related outcomes, with a specific focus on person–organization fit and misfit.

## Limitations and Future Research

This study provides a sound and concise measure of organizational spirituality, validated in a large sample of Polish employees, and demonstrates the scalar measurement invariance of the tool across diverse sociodemographic and work-related variables. Moreover, the significant effect of organizational spirituality on job satisfaction was supported even after controlling for individual spirituality at work and sociodemographic variables.

The key limitation of this study is its cross-sectional design. A longitudinal research approach is needed to examine whether organizational spirituality leads to improvements in employee job satisfaction. Although the sample was well diversified in terms of sociodemographics, the generalizability of the study is limited because most participants identified as Roman Catholics. Further studies are needed to examine the psychometric properties of the OSVS-S in countries with diverse cultural and religious backgrounds.

It is also recommended to investigate the sociodemographic, work-related, and psychological factors that facilitate the development of a workplace culture that promotes organizational spirituality. A promising area for future research that integrates individual- and meso-level workplace spirituality is spiritual leadership (Fry, 2017; see also Syahir et al., 2025). In line with the person–organization fit theory (Kristof, 1996; Kristof-Brown et al., 2005), leaders who implement spiritual values within a spiritually oriented organizational environment can enhance motivation among spiritually inclined employees to express their spirituality at work and serve as role models in shaping spiritually based attitudes among all employees. Further, according to situational strength theory (Meyer et al., 2020), when spiritual leaders and spiritually oriented workplaces work synergistically, they can create a strong situational context that clarifies and reinforces desirable attitudes. This strong context may shape employees’ perceptions and promote the expression of spiritually aligned behaviors.

## Conclusion

The study contributes to the literature by providing a rigorous measurement of workplace spirituality at the organizational level. The short 10-item version of the OSVS demonstrates excellent psychometric properties and is therefore recommended for use in future studies on organizational spirituality. Establishing scalar measurement invariance enables researchers to meaningfully compare perceived levels of organizational spirituality across gender, age, employees’ religiosity levels, job positions, employment sectors, and organization sizes. Moreover, organizational spirituality significantly contributed to job satisfaction after controlling for individual spirituality at work and sociodemographic variables. This result emphasizes the importance of simultaneously including both organizational spirituality and individual spirituality at work in models to control for each other’s effects. Additionally, it highlights the need for interventions aimed at enhancing organizational spirituality by promoting spiritual values throughout the workplace, which can foster a more supportive and meaningful environment and lead to improved employee outcomes.

## Data Availability

The data that support the findings of this study are available from the corresponding author (EC), upon reasonable request.

## Appendix 1: Translation and Polish validation of the Organizational Spiritual Values Scale

The Polish translation and validation of the Organizational Spiritual Values Scale (OSVS) followed the standard procedure described by Koenig and Al Zaben (2021) to ensure conceptual equivalence to the original scale and cultural relevance for the target population. The preparation of the Polish version of the OSVS included the following steps, as outlined by the Professional Society for Health Economics and Outcomes Research (Wild et al., 2005):

1. *Preparation:* Permission was obtained from the developer of the OSVS (the lead author).
2. *Forward translation:* Two independent native Polish speakers fluent in English, both born and raised in Poland and currently residing there, translated the scale.
3. *Reconcilation:* Differences between the translations were discussed by the key in-country researcher and the translators, and a unified version was established.
4. *Back-translation:* The scale was back-translated from Polish to English by two native English speakers fluent in Polish, who served as independent translators. These individuals were different from those who performed the forward translation.
5. *Back-translation review* and *Harmonization* (combined): The back-translated version was compared with the original to identify any discrepancies, which were then resolved through revisions to the Polish version. During a harmonization meeting, the key in-country researcher, together with the forward and back translators, reviewed and compared the Polish and English versions of the scale.
6. *Cognitive debriefing*: Five individuals with diverse sociodemographic characteristics were presented with the translated scale and asked to provide comments on the understandability and appropriateness of the instructions, items, and response categories.
7. *Review of cognitive debriefing:* The key in-country resesarcher introduced revisions in the instruction, items, and response categories, considering the results of cognitive debriefing.
8. *Proofreading:* A final round of quality control was conducted by the key in-country researcher to address any minor errors in language.
9. *Finalization:* A final brief report outlining the step-by-step procedure of the tool validation was prepared.

The validation of the translated scale, which is presented in the body of the manuscript, was performed also in line with the recommendations of Koenig and Al Zaben (2021) and included the following analyses: confirmatory factor analysis (CFA) to assess factor validity, item reduction based on the CFA results, reexamination of the revised model, and evaluation of the scale’s reliability, construct validity, and incremental validity.

## Appendix 2: Descriptive statistics and standardized factor loadings for the original version of the OSVS

**Table:**

Item	*M*	*SD*	Skewness	Kurtosis	Standardized factor loadings	Item included in the OSVS-S?
1	2.65	1.14	−0.01	−0.80	0.57	No
2	2.28	1.10	0.34	−0.83	0.61	No
3	2.53	1.20	0.12	−1.15	0.73	No
4	2.92	1.17	−0.18	−0.87	0.79	Yes
5	3.09	1.17	−0.30	−0.77	0.72	Yes
6	2.65	1.15	0.02	−0.94	0.82	No
7	2.91	1.17	−0.24	−0.89	0.73	No
8	2.93	1.18	−0.17	−0.85	0.79	Yes
9	2.82	1.16	−0.11	−0.87	0.87	Yes
10	2.53	1.18	0.18	−0.91	0.86	No
11	2.95	1.18	−0.21	−0.73	0.81	Yes
12	2.68	1.15	0.03	−0.87	0.76	Yes
13^a^	3.28	1.09	−0.10	−0.59	−0.11	No
14	2.98	1.08	−0.24	−0.55	0.77	No
15	2.86	1.15	−0.14	−0.85	0.81	Yes
16	3.14	1.09	−0.37	−0.52	0.67	No
17	2.89	1.11	−0.24	−0.69	0.80	Yes
18	2.58	1.17	0.09	−0.96	0.84	Yes
19	3.20	1.14	−0.46	−0.50	0.64	No
20	3.28	1.12	−0.51	−0.44	0.67	Yes

*M* = mean, *SD* = standard deviation, OSVS-S = short version of the OSVS.

^a^Responses to negatively worded Item 13 were recoded.

## Appendix 3: Factorial structure of the OSVS

There were no missing values for the variables used in the study. Descriptive statistics for the OSVS items are presented in Appendix 2. The means ranged from 2.28 (Item 2) to 3.28 (Items 13 and 20). The results of the CFA for the 20-item OSVS showed that the model fit was unacceptable, as indicated by the values of the model fit criteria (χ^2^(170) = 1,178.86, χ^2^/df = 6.93, CFI = 0.878, TLI = 0.864, RMSEA = 0.082 (90% CI 0.078, 0.086), SRMR = 0.051). For the negatively worded Item 13, the standardized loading was −0.11. For the remaining items, the standardized loadings ranged from 0.57 (Item 1) to 0.87 (Item 9; see Appendix 2).

To improve the psychometric properties of the measure, Item 13 was first removed from the analysis. A CFA was then repeated, and the values of the modification indices (MI) were inspected. The largest value of MI (167.81) was noted for Items 1 and 2. The wording of these two items was then compared, which revealed the similarity of their content. In terms of factor loadings, Item 1 had a lower factor loading than Item 2 (0.57 vs. 0.61), and it could also be more related to one’s religious beliefs, as suggested by the term “saint,” which is often associated with God/a Higher Power. Thus, Item 1 could be problematic and difficult for nonreligious participants to agree with. As a consequence, this item was removed from further analysis.

The CFA conducted on 18 items showed the largest MI value for Items 10 and 18 (91.60). Both items have similar content, referring to the meaning and purpose of life. The standardized factor loadings were high for both items (0.85 vs. 0.84). The comparison of the total MI values for Items 10 and 18 with all the remaining items of the OSVS showed that the total value of MI was 235.29 for Item 10 and 123.02 for Item 18. Based on this result, Item 10 was removed from further analysis.

In the next round of analysis, the largest MI value (60.75) was noted for Items 5 and 19. The content of both items is very similar, as they both refer to the role of veracity in the organization. Item 5 had a considerably higher factor loading than Item 19 (0.73 vs. 0.66). Based on this, the latter was removed.

In the next analysis, the value of the MI for Items 2 and 3 was noted to be the largest (44.62). Item 2 had a substantially lower factor loading than Item 3 (0.59 vs. 0.71). Moreover, its content is quite abstract and general and thus could be difficult for some participants to understand. Therefore, it was removed from the analysis.

The next pair of items with the largest MI value in the next round of analysis was Item 7 and Item 8 (40.49). The content of both items was similar, as they expressed concerns about caring for others. Item 8 had a higher factor loading than Item 7 (0.79 vs. 0.71). Regarding the remaining items, the total value of MI for Item 7 with other items was higher than the total value of MI for Item 8 with other items (35.25 vs. 20.35). Therefore, Item 7 was eliminated.

In the next round, the highest value of MI (37.24) was noted for Items 6 and 18. Both items are closely related to the meaning of life, although Item 6 is more situation-specific, as it refers to relationships with coworkers as the source of the meaning of life. The comparison of the items’ loadings showed a slight advantage of Item 18 over Item 6 (0.82 vs. 0.80). Moreover, Item 6 yielded a larger total value of MI with the remaining items compared to Item 18 (100.23 vs. 67.1). Based on these results, Item 6 was removed.

Further analysis revealed the largest value of MI for Items 14 and 16 (26.46). These items refer to the way employees interact with each other (i.e., how they react when other people are talking about their own problems and whether they see the opportunity to share their own thoughts with other employees). Since Item 14 had a much higher loading than Item 16 (0.80 vs. 0.68), the latter was removed from the analysis.

In the next round, Items 3 and 18 were selected as having the largest MI (18.10). The comparison of factor loadings indicated the advantage of Item 18 over Item 3 (0.81 vs. 0.70). As a result, Item 3 was eliminated from the measure.

In the last step, the highest MI was noted for Items 9 and 14 (14.05). After comparing the standardized item loadings of the items (0.87 vs. 0.79), Item 14 was removed from further analyses.

At this point, the obtained 10-item version of the OSVS (named the OSVS-S) showed only one large MI (i.e., larger than 10) for residuals of Items 18 and 20. Since the value of 10 was exceeded only marginally (11.10), the shortening of the measure was ended at this point, and the OSVS-S was subjected to further analyses.

## Appendix 4: The short version of the Organizational Spiritual Values Scale (OSVS-S)

### OSVS-S

Wnuk & Charzyńska, 2025

Adapted from the Organizational Spiritual Values Scale (OSVS; Kolodinsky et al., 2008)

We would like to ask you some questions about the general climate in your company. Using a scale from 1 to 5, please answer the following in terms of how it really is in your company, not how you would prefer it to be. Please be as candid as possible, remember, all your responses will remain strictly anonymous.

**Table:**

	1 = Mostly false	2 = Somewhat false	3 = Somewhat true	4 = Mostly true	5 = Completely true
The organization values the relationship among everyone who works here	1	2	3	4	5
Being truthful is important to a successful life in this organization	1	2	3	4	5
This organization is sensitive to the pain and suffering of others	1	2	3	4	5
It is important to this organization that employees are whole and complete people	1	2	3	4	5
In this organization, all forms of life are valuable	1	2	3	4	5
There is an overall sense of sadness when someone in this organization is in pain	1	2	3	4	5
This organization promotes health and inner peace	1	2	3	4	5
The organization encourages us to put the interests of others before our own when making a decision	1	2	3	4	5
In this organization we are encouraged to actively seek a sense of purpose in our lives	1	2	3	4	5
We are encouraged to mentor and help new people entering the organization	1	2	3	4	5

### OSVS-S

Wnuk i Charzyńska, 2025

Adaptacja the Organizational Spiritual Values Scale (OSVS; Kolodinsky i in., 2008)

Zadamy Ci kilka pytań na temat ogólnego klimatu panującego w Twoim miejscu pracy. Używając skali od 1 do 5, ustosunkuj się do poniższych stwierdzeń. Weź pod uwagę to, jak naprawdę jest w organizacji/firmie, w której pracujesz, a nie jak chciałabyś/chciałbyś, by było. Prosimy Cię o szczerość – wszystkie Twoje odpowiedzi pozostaną anonimowe.

**Table:**

	1 = W ogóle nieprawdziwe	2 = Raczej nieprawdziwe	3 = Ani prawdziwe, ani nieprawdziwe	4 = Raczej prawdziwe	5 = Zdecydowanie prawdziwe
1. Ta organizacja/firma docenia relacje, jakie się tworzą między pracownikami	1	2	3	4	5
2. Aby odnieść sukces w tej organizacji/firmie, ważna jest prawdomówność	1	2	3	4	5
3. W tej organizacji/firmie widać wrażliwość na ból i cierpienie innych	1	2	3	4	5
4. Ta organizacja/firma dba o to, by pracownicy byli ludźmi spełnionymi i mieli poczucie kompletności	1	2	3	4	5
5. W tej organizacji/firmie ceni się wszystkie formy życia	1	2	3	4	5
6. Jeżeli ktoś w tej organizacji/firmie cierpi, to panuje ogólne poczucie smutku	1	2	3	4	5
7. Ta organizacja/firma promuje zdrowie i spokój wewnętrzny	1	2	3	4	5
8. Ta organizacja/firma zachęca nas do tego, by przy podejmowaniu decyzji dbać przede wszystkim o dobro innych	1	2	3	4	5
9. W tej organizacji/firmie jesteśmy zachęcani, by aktywnie poszukiwać celu swojego życia	1	2	3	4	5
10. Jesteśmy zachęcani, by wspierać nowe osoby dołączające do organizacji/firmy i im pomagać	1	2	3	4	5

## Appendix 5: Means, standard deviations and correlations for the OSVS-S items

**Table:**

Item no. (OSVS)	Item no. (OSVS-S)	*M*	*SD*	Correlation coefficients									
				4	5	8	9	11	12	15	17	18	20
4	1	2.92	1.17	1									
5	2	3.09	1.17	0.62	1								
8	3	2.93	1.18	0.63	0.54	1							
9	4	2.82	1.16	0.71	0.67	0.68	1						
11	5	2.95	1.18	0.63	0.59	0.68	0.72	1					
12	6	2.68	1.15	0.61	0.49	0.62	0.64	0.63	1				
15	7	2.86	1.15	0.64	0.57	0.64	0.71	0.67	0.62	1			
17	8	2.89	1.11	0.59	0.58	0.63	0.69	0.66	0.59	0.68	1		
18	9	2.58	1.17	0.62	0.55	0.63	0.72	0.65	0.64	0.69	0.69	1	
20	10	3.28	1.12	0.59	0.55	0.52	0.60	0.58	0.49	0.57	0.59	0.50	1
Total score	–	28.98	9.37	0.82	0.76	0.81	0.88	0.84	0.78	0.84	0.82	0.83	0.74

*M* = mean, *SD* = standard deviation. All correlations are significant at a *p*-value < 0.001.

## Appendix 6: Descriptive statistics and correlations between the study variables

**Table:**

Variable	*M*	*SD*	Range	Skewness	Kurtosis	(1)	(2)	(3)	(4)	(5)	(6)	(7)	(8)	(9)
(1) Organizational spirituality	28.98	9.37	10–50	−0.23	−0.48	1								
(2) Gender	47.7%^a^	–	–	–	–	0.00	1							
(3) Age (in years)	42.31	11.99	22–78	0.53	−0.43	0.07*	–0.24***	1						
(4) Religiosity	3.55	1.91	1–7	0.07	−1.22	0.24***	0.07*	0.05	1					
(5) Job position	76.1%^a^	–	–	–	–	0.14***	−0.18***	0.10**	0.04	1				
(6) Employment sector	33.5%^a^	–	–	–	–	−0.01	−0.09*	−0.16***	−0.06	0.05	1			
(7) Organization size	35.5%^b^	–	–	–	–	−0.10**	0.02	−0.07*	−0.14***	−0.01	−0.14***	1		
(8) Individual spirituality at work	7.95	4.56	3–18	0.51	−0.94	0.42***	−0.02	0.10**	0.68***	0.13***	−0.06	−0.14***	1	
(9) Job satisfaction	4.92	1.31	1–7	−0.67	0.57	0.53***	−0.05	0.13***	0.13***	0.12***	0.00	−0.05	0.25***	1

**p* < 0.05, ***p* < 0.01, ****p* < 0.001. *M* = mean, *SD* = standard deviation. Dummy coding was used for gender (0 = men, 1 = women), job position (0 = nonmanagers, 1 = managers), and employment sector (0 = public, 1 = private). Data from a participant who chose the response “other” on the item concerning gender was not included in this analysis. For the employment sector, due to the low number of non-governmental organization employees (*n* = 18), only the public and private sectors were included.

^a^For nominal variables, the percentage for the first category is presented.

^b^For organization size, the percentage for the most frequent response (i.e., 250 employees and more) is presented.
